# Effect of Self-Controlled Feedback on Motor Learning and Its Association with Metacognitive Ability

**DOI:** 10.3390/bs16040517

**Published:** 2026-03-30

**Authors:** Kazuto Yamaguchi, Ryohei Yamamoto, Jun Yabuki, Wataru Nakano, Kazunori Akizuki

**Affiliations:** 1 Department of Rehabilitation, Nihon Institute of Medical Science, 1276 Shimogawara, Moroyama, Saitama 350-0435, Japan; 2 Department of Rehabilitation, Kumamoto Health Science University, 325 Izumimachi, Kita-ku, Kumamoto 861-5533, Japan; yamamoto.rh@kumamoto-hsu.ac.jp; 3 Department of Rehabilitation, School of Health Sciences, Tokyo University of Technology, 5-23-22, Nishikamata, Ota-ku, Tokyo 144-8535, Japan; yabukijn@stf.teu.ac.jp; 4 Department of Shizuoka Physical Therapy, Faculty of Health Science, Tokoha University, 1-30 Mizuochi, Aoi-ku, Shizuoka 420-0831, Japan; w-nakano@sz.tokoha-u.ac.jp; 5 Department of Physical Therapy, Mejiro University, 320 Ukiya, Iwatsuki-ku, Saitama 339-8501, Japan; k.akizuki@mejiro.ac.jp

**Keywords:** self-control, motor learning, metacognition

## Abstract

Self-controlled feedback promotes motor skills among patients with bodies altered by disease or injury. However, mechanisms underlying its effectiveness remain unexplored. This study determined whether metacognitive ability is involved in the learning benefits of self-controlled feedback during motor skill acquisition. Twenty-eight healthy adults (14 women; mean age = 21.6 ± 0.5 years), assigned to a self-control group (which received knowledge of the results only when requested) or a yoked group (which received knowledge of the results with the paired participants in the self-control group), performed a golf putting task. The experiment comprised a pre-test, practice trials, and a retention test administered 24 h after practice completion. Metacognitive ability was assessed after practice using the Adult Metacognition Scale. The self-control group showed greater improvement from the pre-test (V = 4.5, *p* < 0.01) and scored higher than the yoked group on the retention test (U = 51, *p* = 0.02). No between-group differences were found for any metacognitive subscale scores. Metacognitive monitoring was positively correlated with putting performance improvement only in the yoked group (*p* < 0.05, r = 0.56). The self-control group showed enhanced motor learning compared with the yoked group. Metacognitive monitoring was associated with learning only when feedback timing was externally determined, suggesting that self-control benefits learners with lower metacognitive monitoring.

## 1. Introduction

In rehabilitation settings, patients with bodies altered by disease or injury must acquire new motor skills; that is, they must engage in motor learning. A wide range of practice conditions thought to facilitate motor learning have been examined, including practice schedules (Czyż et al., 2024; Donovan & Radosevich, 1999), augmented feedback (Petancevski et al., 2022; Sigrist et al., 2013; Yabuki et al., 2022), task difficulty (Akizuki & Ohashi, 2015; Akizuki et al., 2025b), attentional focus (Hawkins et al., 2025; Wulf, 2013), and physical guidance (Heuer & Lüttgen, 2015; Marchal-Crespo et al., 2015; Yamaguchi et al., 2020). Among these factors, “self-control,” whereby learners actively participate in shaping their own practice environment, has repeatedly been reported to enhance motor learning (Sanli et al., 2013; Wang et al., 2025). Janelle et al. (1995, 1997) first tested the effect of self-control on the acquisition of motor skills. In a target-throwing task, they reported that when learners could control the provision of performance knowledge on their throwing form, they showed smaller errors in the retention test than groups that did not control knowledge provision (Janelle et al., 1997). Beyond the self-control of augmented feedback (Akizuki et al., 2025a; Drews et al., 2024; Pourazar et al., 2023; Yamamoto et al., 2025), self-control has also been studied in terms of choices such as the task difficulty of practice (Andrieux et al., 2012), number of practice repetitions (Aiken et al., 2020; Post et al., 2011), practice schedule (Keetch & Lee, 2007), and use of physical assistance (Williams et al., 2017; Wulf & Toole, 1999).

Although many studies support the benefits of self-control, other reports also show that self-control has no or only a small additional effect on motor learning (McKay et al., 2022b; St Germain et al., 2023). However, the mechanism underlying the effectiveness of self-control has not yet been elucidated. Drews et al. (2024) focused on the frequency of self-controlled feedback requests and found no learning benefit for learners who requested feedback more frequently. Ma et al. (2025) focused on task difficulty and reported that self-control enhanced learning when the task was difficult but not when the task was easy. Moreover, a meta-analysis by McKay et al. (2022a) suggested that, after correcting for publication bias, the effect of self-control on motor learning was very small. According to previous research, self-control may not always be effective for motor learning. Its effectiveness may depend on the practice environment and the learning strategy of the learner.

Chiviacowsky and Wulf (2002) compared the timing of self-controlled feedback by dividing it into choices made before and after practice trials, and showed that post-trial self-control produced greater learning benefits than pre-trial self-control. This finding suggests that the benefit of self-control increases when learners reflect on a recently completed task and decide whether feedback is necessary. Therefore, reflection on one’s own performance, that is, metacognition, can be considered crucial for effective self-controlled feedback.

Metacognition has been defined as “the ability to reflect upon, understand, and control one’s learning” (Schraw & Dennison, 1994, p. 460). Furthermore, metacognition can be divided into knowledge (“knowledge or beliefs about how one learns”), monitoring (“assessing the current state of learning or performance”), and control (“regulating some aspect of learning”; Rivers et al., 2020, p. 550). In self-controlled feedback, learners engage in metacognitive monitoring by reflecting on their own performance and deciding whether feedback is needed. Metacognitive control is considered to operate when formulating a motor plan for the next trial after feedback (or even in its absence). Metacognitive knowledge refers to an understanding of the characteristics of learning and is presumed to operate throughout practice. Carter et al. (2014) reported that, even in yoked groups, having learners reflect on their own performance and completing an error-estimation task after each trial led to improved motor learning. Although the metacognitive ability was not directly assessed, these findings suggest that engaging in metacognition (in this case, monitoring) during practice can facilitate motor learning.

Taken together, these results suggest that the effectiveness of self-controlled feedback on motor skill learning is influenced by the learner’s metacognitive ability and the exertion of that ability during practice; however, the precise nature of this relationship remains unclear. Therefore, the present study aimed to determine whether metacognitive ability is involved in the effects of self-controlled feedback on motor learning where performance is inherently variable and learners must interpret errors under uncertainty. We hypothesized that, in the self-controlled condition, learners who actively engage metacognitive processes during practice (i.e., monitoring and control when deciding whether feedback is needed) would show greater learning benefits than learners who do not. In contrast, in the yoked condition—where learners do not control feedback delivery—we expected learning outcomes to be more strongly determined by individual differences in trait metacognitive ability. The findings of this study can clarify the conditions under which self-controlled feedback effectively facilitates motor skill learning, as well as those under which such benefits may be limited or absent.

## 2. Materials and Methods

### 2.1. Participants

A power analysis was performed using G*Power 3.1 (Heinrich Heine University, Düsseldorf, Germany) to calculate the required sample size for a mixed analysis of variance (ANOVA) with repeated measurements. Based on a previous study (Pourbehbahani et al., 2023) that employed a practice condition and motor task similar to those in our study, the power analysis was conducted using the following values: α = 0.05, statistical power = 0.80, effect size (Cohen’s f) = 0.59, number of groups = 2, and number of repeated measures = 2. The analysis revealed that a minimum sample size of 26 participants was required. Considering the possibility of dropouts during the experiment, 28 participants (mean age = 21.6 years, SD = 0.50; 14 women, 14 men) were recruited and completed this experiment. None of the participants had prior experience with the experimental task and were unaware of the specific study purpose. They did not report any neurological or vestibular disorders or orthopedic conditions before participating in this study. Written informed consent was obtained from all participants prior to the experiment. The study protocol was approved by the Institutional Review Board of Mejiro University (approval number: 28-14).

### 2.2. Task and Apparatus

**Figure 1:**
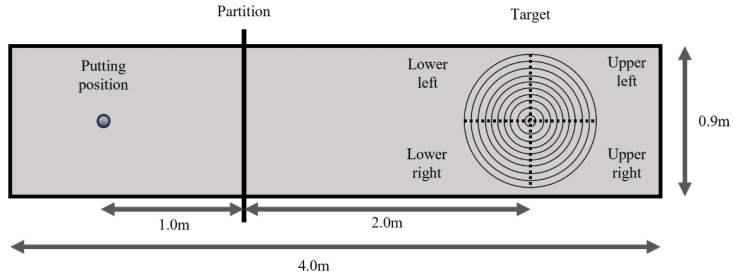
Schematic of the motor task.

The task required participants to putt a golf ball (diameter of 4 cm) on a target placed 3 m from the participant. Ten concentric circles were drawn around the center of the target at 4-cm increments (radii: 4, 8, 12, …, 40 cm), and points were assigned according to the ball’s distance from the center (10, 9, 8, 7, 6, 5, 4, 3, 2, and 1). If the golf ball was outside the circle with a radius of 40 cm, the participant was given zero points. We set a partition 1 m apart from the hitting position to avoid observing the results (Figure 1). Before the task, participants were informed of the target configuration and the scoring rules and were instructed to aim for the center of the target and to perform as accurately as possible.

### 2.3. Metacognitive Abilities

We used the Adult Metacognition Scale (AMS) developed by Abe and Ida (2010) to assess learners’ metacognitive abilities. The AMS was developed based on the Metacognitive Awareness Inventory, which was originally developed by Schraw and Dennison (1994). The AMS consists of three factors (monitoring, control, and knowledge), and twenty-eight items are rated on a 5-point Likert scale (1 = “strongly disagree,” 5 = “strongly agree”). Higher AMS scores indicate higher metacognitive ability.

### 2.4. Procedure

Twenty-eight participants were allocated to two groups: self-control (*n* = 14) and yoked (*n* = 14). Allocation was constrained to match sex distribution between groups; participants in the yoked group were sex-matched to those in the self-control group. In the self-control group, the participants received knowledge of results (KR) for the trial that they requested. The KR was the position and point from the target (e.g., six points on the upper right). In contrast, the yoked group received KR on the same schedule as the paired self-control group participants. Under these conditions, although the timing and frequency of KR were identical between the self-control and yoked groups, KR was provided contingent upon each learner’s individual performance. Prior to the experiment, the participants performed several familiarization trials. Subsequently, a pre-test consisting of 10 trials was conducted. In the pre-test, KR was not provided in all conditions. Practice was conducted three minutes after the pre-test ended, which consisted of five blocks that involved 10 practice trials; that is, all practice trials included 50 trials. The inter-block interval was set to one minute. In the self-control group, participants were told that they could request KR after each trial, and it was provided if they wanted it. After the practice session, we quantitatively measured participants’ metacognitive abilities using the AMS. Twenty-four hours after the practice session, a retention test identical to the pre-test was conducted.

### 2.5. Data Analysis

We used putting points of the motor task and the average of each of the three subcategories of the AMS for the analysis. Prior to inferential testing, all variables were tested for normality using the Shapiro–Wilk test within each group and time point; *p* < 0.05 was interpreted as evidence against normality. Based on the results of the normality tests, parametric procedures were used when the normality assumption was met, whereas nonparametric procedures were used when normality could not be assumed. A 2 (groups: self-control, yoked) × 5 (blocks × 10 trials) ANOVA with repeated measures on the last factor was used to analyze the accuracy data for the practice phase. When significant main effects or interactions were observed in the two-way ANOVA, a post hoc analysis (Bonferroni method) was conducted. On the other hand, because normality could not be assumed for the test period (*p* < 0.05), the nonparametric tests described below were employed and *p*-value adjustments were applied. The Mann–Whitney U test was performed to examine differences in the practice conditions (self-control and yoked) in the pre-test and retention test. The Wilcoxon signed-rank sum test was performed to examine changes from the pre-test to the retention test for each condition. The *p*-values were corrected using Holm’s method. To examine the differences between the self-control and yoked groups in terms of metacognitive ability, a t-test was used for each of the three AMS subcategories (knowledge, monitoring, and control). Pearson’s correlations were used to examine the relationships between the three AMS subcategories and the degree of change from the pre-test to the retention test of the putting points (retention test − pre-test). For statistical analysis, R version 4. 2. 2 (R Core Team, Vienna, Austria) and SPSS version 29 (IBM Corp., Armonk, NY, USA) with a significance level of 5% were used.

## 3. Results

**Figure 2:**
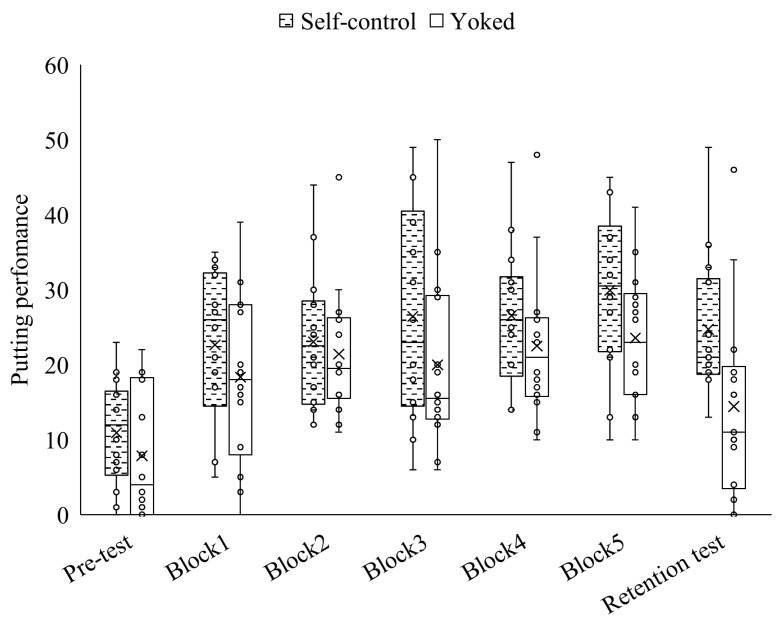
Median putting scores (points) of the practice groups. The solid line indicates the median and the cross indicates the mean. The results of the putting scores from the pre-test to the retention test have been shown. The box plot shows the medians and interquartile ranges. Whiskers extend to the lowest and highest observations within ± 1.5 × interquartile ranges of the quartiles. The Mann–Whitney U test revealed that the self-control group had significantly higher retention test scores than the yoked group. The Wilcoxon signed-rank sum test showed that the retention test scores significantly improved from the pre-test in self-control group.

The results of the test and practice trials for each group are shown in Figure 2. In the self-control group, participants requested feedback an average of 4.9 times during the practice session. In addition, the mean number of feedback requests by block is provided in Table 1.

In the practice session, the main effect of practice condition [*F*(1, 26) = 1.76, *p* = 0.14, *η_p_*^2^ = 0.06], the main effect of block [*F*(4, 104) = 0.31, *p* = 0.87, *η_p_*^2^ = 0.01], and the interaction between group and block [*F*(4, 104) = 0.31, *p* = 0.87, *η_p_*^2^ = 0.01] were not significant. Holm-adjusted *p*-values are reported. The self-control group demonstrated significantly greater improvement from the pre-test to the retention test (*V* = 4.5, *p* < 0.01, *r* = 0.55), whereas the yoked group did not show significant improvement (*V* = 27.5, *p* = 0.25, *r* = 0.22). Furthermore, although there was no difference between the groups on the pre-test (*U* = 18, *p* = 0.26, *r* = 0.21), the self-control group scored significantly higher than the yoked group on the retention test (*U* = 51, *p* = 0.02, *r* = 0.44). No differences were observed between the self-control and yoked groups in any subcategory of the AMS (*p* > 0.05; Table 2).

Correlations among the three independent factors of the AMS and the degree of change from the retention test to the pre-test of the putting points are presented in Table 3.

Only the yoked group, the monitoring category of the AMS, had a significant positive correlation with the degree of change in performance (*p* = 0.04, *r* = 0.56).

## 4. Discussion

This study aimed to determine whether metacognitive ability is involved in the effects of self-controlled feedback use on motor learning. Consistent with many prior studies, the self-control group outperformed the yoked group on the retention test, indicating a learning benefit. In contrast, there were no significant between-group differences in metacognitive ability between the self-control and yoked groups. Notably, in the yoked group alone, metacognitive monitoring showed a significant positive correlation with learning outcomes. These results indicate that learners’ metacognitive abilities influenced their learning outcomes in the yoked group.

We hypothesized a positive correlation between learning outcomes and metacognitive abilities for both the self-control and yoked groups. However, contrary to this, no such association was found between the learning outcomes of the self-control group and metacognitive abilities in any AMS subcategory. Metacognitive monitoring was positively correlated with learning outcomes only in the yoked group. Metacognitive monitoring is defined as “assessing the current state of learning or performance” (Rivers et al., 2020, p. 550), that is, learners’ reflection on their performance after each practice trial. In the yoked group, learners who engaged more in such reflection exhibited greater learning benefits. Barros et al. (2019) reported that when learners in the yoked condition were required to perform error estimation (i.e., reflection on their own performance), they showed greater learning benefits than the self-control group. Therefore, metacognitive monitoring during practice may account for the learning benefits typically attributed to self-control. In our study, learners in the yoked group who did not decide whether to receive feedback may have differed in the extent to which they reflected on their performances. Those with higher metacognitive monitoring likely reflected more, whereas those with lower monitoring likely reflected less. This difference underpins the correlation observed between metacognitive monitoring and motor learning in the yoked group.

In the self-control group, no association was observed between learning outcomes and metacognitive abilities. Previous studies have shown that learners in self-controlled conditions tend to request feedback when they feel they are performing well (Chiviacowsky & Wulf, 2002; Fairbrother et al., 2012). This implies that such learners reflect on their performance when deciding whether to receive feedback. Furthermore, studies comparing the timing of self-control (before vs. after a trial) have demonstrated that learners exhibit greater learning when they assess their need for feedback after a trial (Carter et al., 2014; Chiviacowsky & Wulf, 2005). This finding suggests that reflecting on one’s performance is essential for effective self-control. In the present study, the practice condition in the self-control group likely provided opportunities for reflection, regardless of individual differences in metacognitive abilities. Consequently, no association was observed between metacognitive ability and learning outcomes. This does not necessarily indicate that reflection was unimportant in the self-controlled condition; rather, the self-control procedure may have encouraged most learners to engage in reflection when deciding whether feedback was needed, thereby reducing the extent to which individual differences in trait monitoring were expressed in learning outcomes. In other words, the variability in learning attributable to trait-level monitoring may have been attenuated (i.e., reducing variability) in the self-controlled conditions. Therefore, self-controlled feedback was effective regardless of metacognitive monitoring ability level.

This pattern of results can be interpreted within the two theoretical frameworks proposed to explain the advantages of self-control in motor learning: a motivational perspective (Lewthwaite et al., 2015; Wulf & Lewthwaite, 2016) and an information-processing perspective (Carter et al., 2014; Chiviacowsky & Wulf, 2005). According to Wulf and Lewthwaite’s (2016) OPTIMAL theory, self-control enhances learners’ autonomy, thereby increasing their expectations of success and intrinsic motivation. This motivation has been proposed to improve performance and motor learning. Empirical support for this motivational perspective includes findings showing that self-controlled practice elements unrelated to the motor task (specifically, the color of the ball; Lewthwaite et al., 2015, or the mat placed under the target; Wulf et al., 2018) can facilitate motor learning. In contrast, the information-processing perspective proposes that self-control increases engagement with cognitive processes, resulting in deeper information processing. Supporting evidence includes reports showing that self-control leads to deeper processing of feedback (Grand et al., 2015) and improves learners’ error-estimation abilities (Carter et al., 2014). In the yoked group, which was the control condition in this study, learning outcomes were correlated with learners’ metacognitive monitoring. However, in the self-control group, high learning outcomes were achieved regardless of the metacognitive monitoring ability. Furthermore, no significant differences were found between the self-control and yoked groups in any metacognitive ability subcategory. This suggests that the opportunity for self-controlled feedback in the self-control group had an effect that compensated for the learners’ metacognitive monitoring abilities. Although the present study did not aim to determine which explanatory perspective is more appropriate, the results partially support the information-processing perspective as a mechanism underlying the effects of self-control.

This study had some limitations: we comprehensively assessed learners’ metacognitive abilities and examined their association with motor learning. However, we did not directly measure whether learners reflected on their performance during practice, which limits our ability to test mechanistic predictions about task behavior. In addition, we did not include any direct, process-level measures of online metacognitive engagement during practice. Therefore, future research should evaluate the extent to which metacognition is exercised during practice in both self-controlled and yoked conditions, in addition to measuring metacognitive abilities. In addition, although we experimentally manipulated the practice condition (self-controlled vs. yoked feedback), this manipulation did not result in a detectable between-group difference in metacognitive questionnaire scores. Therefore, the correlational findings should not be interpreted as evidence that metacognitive ability causally determines motor learning outcomes. Moreover, the correlational analyses were based on a relatively small sample (*n* = 14 per group), and thus these results should be considered exploratory and interpreted with caution. Furthermore, there were no differences in metacognitive abilities between the self-control and yoked groups. We assessed learners’ metacognitive abilities after the practice trials but were unable to conduct pre-practice assessments. Consequently, we were unable to detect changes in the learners’ metacognitive abilities. Previous studies have reported that learners’ motor learning is enhanced by self-control during practice (Barros et al., 2019) and that long-term educational interventions improve metacognitive abilities (Altıok et al., 2019; Chan et al., 2021). Future research should clarify the effects of long-term exposure to self-controlled practice conditions and the relationship between changes in metacognitive abilities and learning outcomes.

In summary, we attempted to explain the learning benefits of self-controlled feedback in terms of metacognitive ability. Our findings suggest that metacognition, particularly monitoring, influences motor learning outcomes. Although we did not observe a between-group difference in metacognitive questionnaire scores, self-controlled feedback may reduce the extent to which individual differences in metacognition are reflected in learning outcomes, making it an effective strategy for a wide range of learners. In other words, the present findings support an information-processing perspective of the benefits of self-controlled feedback. From a clinical perspective, self-controlled feedback may be a promising and effective learning strategy for learners with lower metacognitive abilities.

## Figures and Tables

**Table 1:** Number of feedback requests in self-control.

	Block 1	Block 2	Block 3	Block 4	Block 5
Mean	5.2	5.2	5.3	4.4	4.6
(SD)	(2.6)	(3.0)	(2.3)	(2.4)	(2.6)

**Table 2:** Average points of three subcategories of the Adult Metacognition Scale in the self-control and yoked groups.

AMS Subcategory	Self-Control Group *n* = 14	Yoked Group *n* = 14	*p*-Value	Effect Size
	Mean ± SD			*r*
Monitoring	2.84 ± 0.41	2.75 ± 0.64	0.67	0.08
Control	3.27 ± 0.42	3.46 ± 0.54	0.32	0.19
Knowledge	3.62 ± 0.50	3.66 ± 0.31	0.82	0.04

*Note:* AMS, Adult Metacognition Scale.

**Table 3:** Pearson’s correlation coefficient between the Adult Metacognition Scale and the degree of change from the retention test to the pre-test of the putting points.

Group	Monitoring	Control	Knowledge
Self-control	−0.23 (0.44)	−0.13 (0.66)	−0.07 (0.82)
Yoked	0.56 (0.04)	0.36 (0.21)	0.14 (0.63)

*Note:* Values are Pearson correlation coefficients; *p*-values are shown in parentheses.

## Data Availability

The datasets generated and analyzed during the current study are available in the Zenodo repository, https://zenodo.org/records/18233372 (accessed on 24 March 2026).

## Abbreviations

The following abbreviations are used in this manuscript:

AMS	Adult Metacognition Scale
KR	Knowledge of results
